# Long-read genome sequencing resolves a de novo complex 18q12.1q21.2 triplication causing partial tetrasomy and reveals its underlying mechanism

**DOI:** 10.1007/s00439-026-02855-0

**Published:** 2026-07-04

**Authors:** Serena Redaelli, Viviana Tritto, Eleonora Mangano, Roberta Bordoni, Elena Sala, Nicoletta Villa, Francesca Crosti, Leda Dalprà, Luigina Spaccini, Alessandra Sirtori, Giovanni Cazzaniga, Donatella Conconi, Angela Bentivegna, Paola Riva

**Affiliations:** 1 School of Medicine and Surgery, University of Milano-Bicocca, Monza, 20900 Italy; 2 Department of Medical Biotechnology and Translational Medicine (BIOMETRA), University of Milan, Milan, 20122 Italy; 3 Institute for Biomedical Technologies (ITB), National Research Council (CNR), Segrate, 20054 Italy; 4 UC Medical Genetics, Fondazione IRCCS San Gerardo dei Tintori, Monza, 20900 Italy; 5 Clinical Genetics Unit, Department of Obstetrics and Gynecology, Vittore Buzzi Children’s Hospital, University of Milan, Milan, 20122 Italy; 6 Scuola di Specializzazione in Genetica Medica, University of Milano-Bicocca, Monza, 20900 Italy

## Abstract

Chromosomal triplications are rare structural variations often associated with complex phenotypes. We report the molecular characterization of a novel intrachromosomal triplication at 18q12.1q21.2 identified in a fetus with ultrasound abnormalities. Conventional karyotyping and array-CGH revealed a partial tetrasomy and a 26 Mb region of loss of homozygosity (LOH), extending from the triplication to the telomere. Long-read sequencing (LRS) identified breakpoint junctions revealing a direct-inverted-direct triplication structure. Breakpoint analysis suggested that this rearrangement arose through a U-type exchange between sister chromatids, likely mediated by microhomology-based mechanisms. This process likely generated a transient dicentric chromosome that subsequently broke during mitosis. The resulting duplicated chromosome may have been stabilized by telomere capture, consistent with the triplicated 18q12.1q21.2 region followed by the 18q21.2q23 LOH. Nine genes within the triplicated region, including *SMAD2* and *SMAD4*, showed high predicted sensitivity to increased dosage, possibly contributing to the clinical phenotype. This study highlights the utility of LRS in defining complex chromosomal rearrangements and emphasizes the importance of molecular breakpoint analysis for understanding pathogenic mechanisms and improving genetic prenatal diagnosis.

**Supplementary Information:**

The online version contains supplementary material available at 10.1007/s00439-026-02855-0.

## Introduction

The routine introduction of Chromosomal Microarray Analysis (CMA) in prenatal and postnatal karyotyping has dramatically changed the field of fine identification of quantitative minute chromosomal changes, such as microdeletions and microduplications, which are too small to be detected with a cytogenetic approach. Even though CMA significantly improves upon conventional cytogenetics for diagnostic investigation, it has a key limitation: it cannot identify balanced chromosomal aberrations.

A group of these variants is represented by complex de novo triplications leading to large genomic alterations (complex chromosomal rearrangements, abbreviated as CCR or CGR). This rearrangement involves three or more DNA breakpoints and/or comprise structures formed by more than one “simple” structural rearrangement occurring simultaneously or in close succession.

Here, we focus on an intrachromosomal triplication leading to partial tetrasomy. Although this chromosomal aberration is considered a rare event, it has been already reported for different chromosomes, and is associated to complex phenotypes (Carvalho et al. 2011). Generally, inverted genomic segments and complex triplication rearrangements are mediated by inverted repeats in the human genome (Grochowski et al. 2024).

Wang et al. in 1999 reported an intrachromosomal triplication of 2q11.2-q21 in a severely malformed infant (Wang et al. 1999). The same authors, reviewing cases reported in the literature with intrachromosomal triplication, highlighted as many as seven of 12 cases involved chromosome 15q11-q13, suggesting that 15q is particularly prone to this kind of rearrangement (Wang et al. 1999). The triplication event was well described for the *MECP2* duplication syndrome (MRXSL, MIM: 300260), a developmental disease caused by copy number variants (CNVs) involving the *MECP2* gene at Xq28, with 100% penetrance. Approximately 26% of individuals with MRXSL syndrome (MRXSL, MIM: 300260) harbor a triplication at Xq28, including the *MECP2* gene in a hemizygous state (Carvalho et al. 2009). In 2023, Pelgrims et al. described four patients showing triplication of the 1p36.3 region, which is known to be responsible for the corresponding syndrome (Pelgrims et al. 2023). Chromosome 1 has also been found to be involved in triplications in another region, 1q21, where both deletions and duplications have been associated with clinical phenotype (Bourgois et al. 2024).

These genomic rearrangements are particularly interesting for their potential impact on gene expression and clinical consequences. Thus, it is important to precisely identify their genomic architecture at the molecular level to support further studies aimed at identifying deregulated genes, dosage sensitive genes, and the potential correlation between the clinical phenotype and the differentially expressed genes. Only a few studies have provided models for the mechanisms generating complex rearrangements, validated at the molecular level (Carvalho et al. 2016).

The recent application of long-read sequencing (LRS) to unravel the architecture of complex genomic regions (mostly constituted by tandem repeats) allows the researchers to identify the breakpoint (BP) junctions of CCRs, uncover the molecular mechanisms leading to derivative chromosomes, and no less important, define the genomic context and the genes involved in the rearrangements (Showpnil et al. 2024; Emiliani et al. 2025).

Here, we report the molecular characterization of 18q12.1q21.2 tetrasomy identified in a fetus with ultrasound abnormalities, which, to our knowledge, has not been previously described. We initially hypothesized the mechanism according to similar rearrangements, and then we validated it at molecular level by LRS. Thanks to this approach, it was possible to identify genes potentially affected by expression dysregulation, sensitive to gene dosage, and consequently associated with the clinical phenotype.

## Results

### Clinical description and post-mortem examination findings

A 29-year-old woman (first pregnancy) sought prenatal diagnosis due to increased fetal nuchal translucency (3.9 mm) at echographic examination along with a reversed ventricular septal defect and tricuspid valve insufficiency. The combined test (PAPP-A and free beta-HCG) resulted in a high risk for trisomy 21 (1:12). Therefore, a chorionic villus sampling was performed at 12 weeks of gestational age. Following the conventional and molecular karyotype results, the pregnancy was terminated voluntarily at 16 weeks and 4 days of gestation and the autopsy was performed. The measurements and the morphological appearance of the organs were consistent with those of a female fetus at gestational age of 15–17 weeks. External inspection revealed mild facial dysmorphisms, characterized by hypertelorism and low-set ears. The most significant finding was a left-sided Congenital Diaphragmatic Hernia (CDH), with intrathoracic herniation of the liver, spleen, and stomach. Examination of the brain revealed features suggestive of neuronal heterotopia involving the parietal lobe of the right hemisphere, and possible focal defects of corticogenesis.

### Conventional and molecular karyotype

**Fig. 1:**
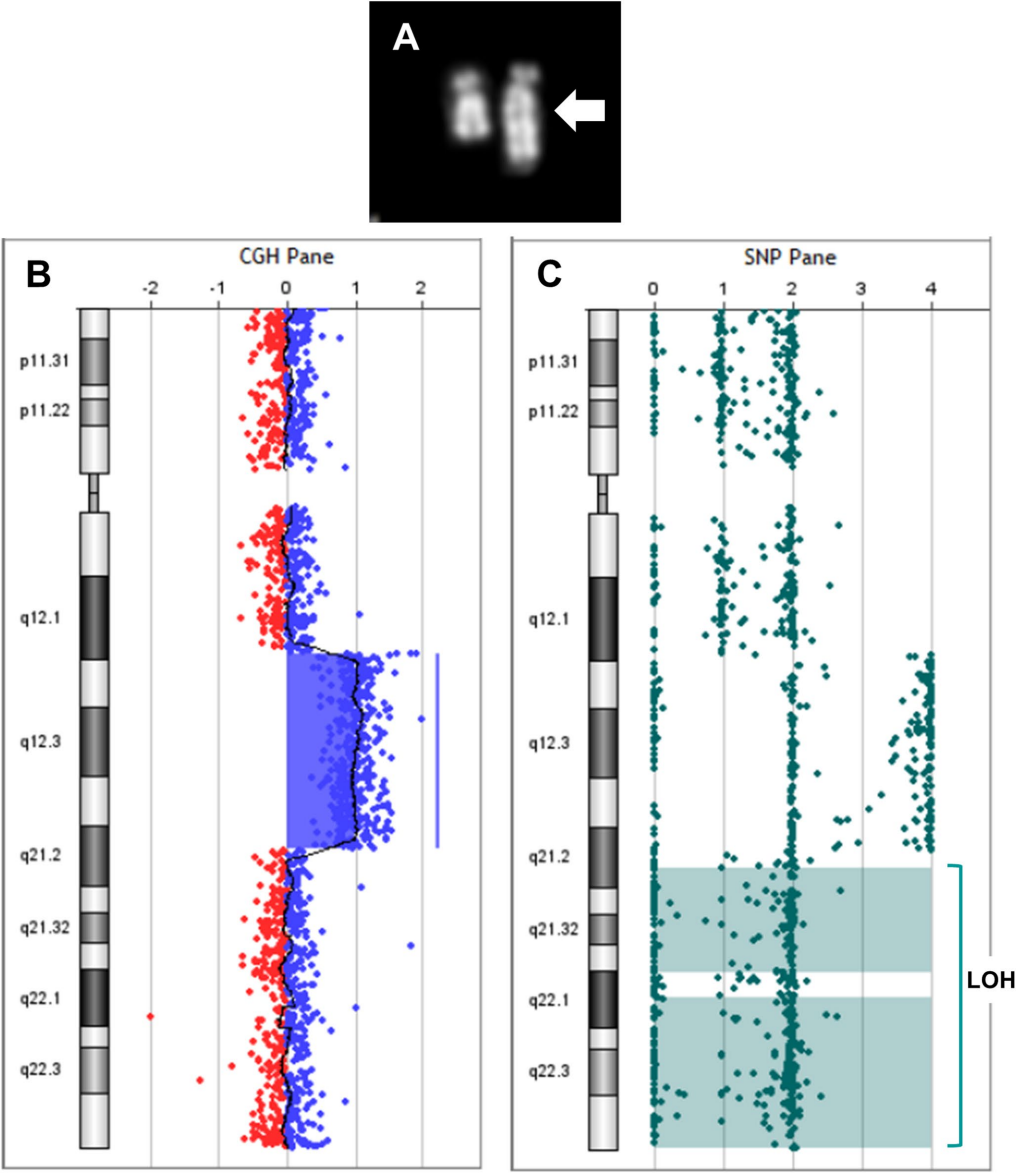
Cytogenetics and molecular karyotype characterization. A: QFQ banded foetal chromosomes 18; the arrow shows the abnormal one. B-C: CGH+SNPs-array results: blue area shows the tetrasomic region in 18q12.1q21.2 (from nt 34,509,207 to nt 52,782,563); aqua green area shows loss of heterozygosis (LOH) in 18q21.2q23 (from nt 54,447,020 to nt 80,221,686)

Cytogenetic analysis performed on both cytotrophoblasts and cultured mesenchymal cells showed a female karyotype with an abnormal chromosome 18, defined as a duplication of region q12.2q22. The duplication was observed in 11 metaphases from cytotrophoblast, whereas 3 cells showed a normal karyotype (46,XX,?dup(18)(q12.2q22)[11]/46,XX[3]). Given the extensive experience of our center and the rigorous cleaning protocol employed, MCC is considered statistically negligible (0.01%) in our direct preparations. Therefore, the 3/14 normal cells observed in the direct preparation most likely represent low-level confined placental mosaicism (CPM) or a monochorionic vanishing twin. Notably, short tandem repeat (STR) analysis on genomic DNA (gDNA) from cultured mesenchymal cells, where maternal cells would have had a greater opportunity to proliferate, conclusively excluded MCC. Interestingly, both conventional and molecular karyotypes performed on cultured mesenchymal cells failed to identify the normal cell line. Array-CGH analysis further validated the existence of the triplicated region:

arr[GRCh38] 18q12.1q21.2(34509207_52782563)x4dn,18q21.2q23(54447020_80221686)x2hmz. Since one chromosome 18 had a normal morphology and the other one was elongated, it is evident that three doses are located on the same chromosome 18 (Fig. 1A, B). Surprisingly, a 26-Mb region, extending from the distal breakpoint of the triplicated region to the 18q telomere, exhibited a loss of heterozygosity (LOH) (Fig. 1C).

### Molecular characterization of tetrasomy 18q12.1q21.2 by LRS

Genomic DNA (gDNA) purified from cultured mesenchymal cells, previously analyzed by array CGH, was subsequently analyzed by LRS to delineate the precise BP junctions and the orientation of the triplicated 18q12.1q21.2 region.

**Fig. 2:**
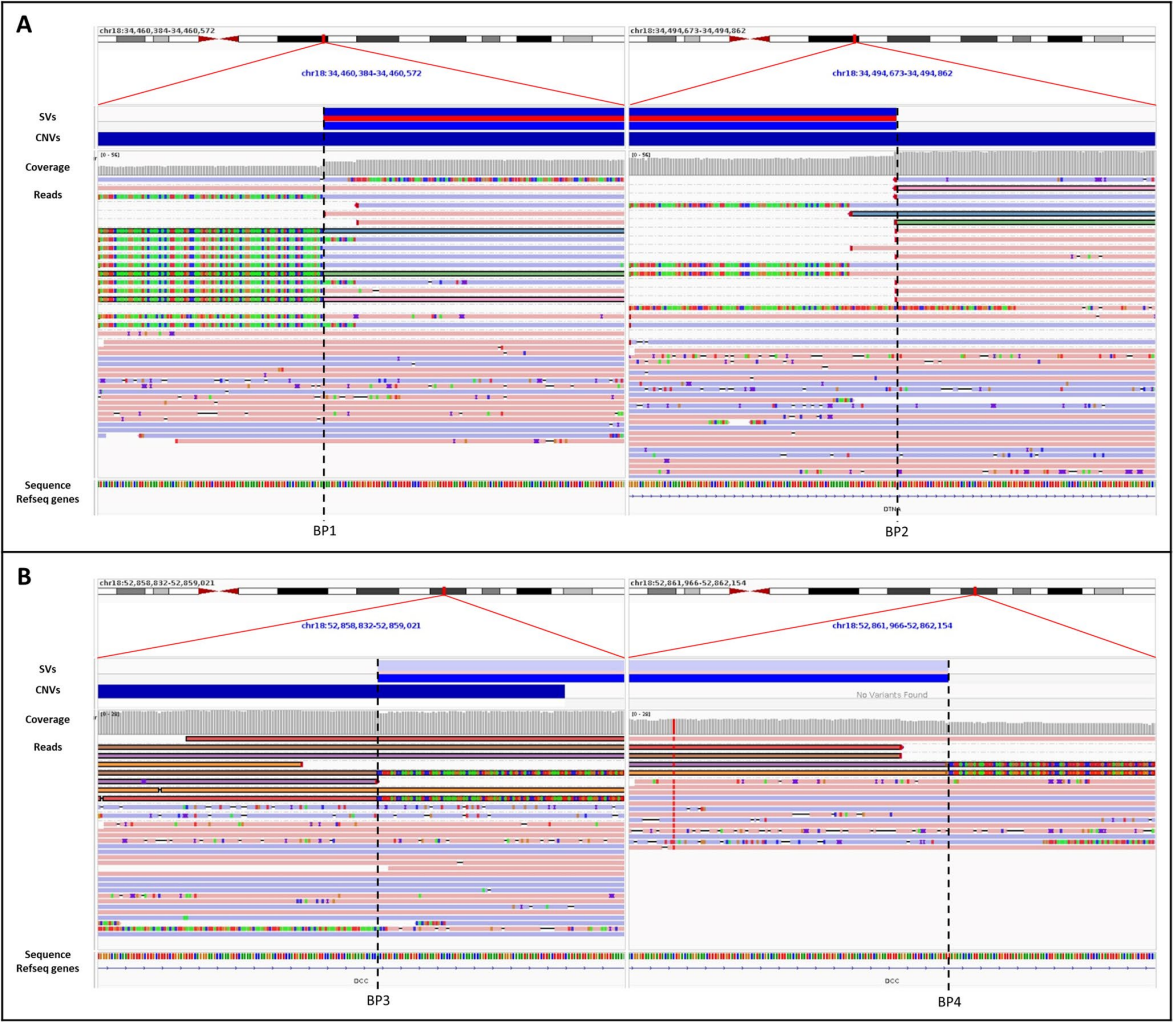
Identification of the junction fragments of 18q12.1q21.2 triplicated region by LRS. The Integrative Genomics Viewer (IGV) visualization shows reads alignments around the 18q inversions’ BPs detected by LRS. The chimeric reads spanning BPs were highlighted by the presence of a misaligned colored end. **A** The read alignment revealed the proximal BP1 and BP2 of the junction fragment, mapping in the 18q21.2 cytogenetic band, between the middle and the last repeats of the CNV. **B** The read alignment identified the distal BP3 and BP4 of the junction fragment, between the first and the second CNV repeats in the 18q12.1

LRS confirmed the previously identified triplication of the 18q12.1q21.2 region. The results showed that the chromosome 18 CNV ranges approximately from 34,460,000 bp to 52,859,000 bp (GRCh38/hg38), for a total size of 18.4 megabases (Table S1). Moreover, two inversions were detected at the BP junctions’ regions (Table S1). Analysis of the alignment between the reads supporting the inversions and the reference genome revealed that the start and end points of both inversions turned out to be the BPs of the two junction fragments between the middle repeat of the 18 Mb CNV and the two flanking repeats (Fig. 2).

Specifically, the BPs of the first inversion were defined by chimeric reads mapping from position chr18:34,460,465 (on the forward or reverse DNA strand), to chr18:34,494,769 on the opposite DNA strand (Fig. 2A). These genomic coordinates represent the proximal BP1 (chr18:34,460,465) and BP2 (chr18:34,494,769), which are located at the junction between the second and third copies of the CNV. The BPs of the second 18q inversion were identified by chimeric reads spanning up to chr18:52,858,932, on one strand, and up to chr18:52,862,079, on the opposite strand (Fig. 2B). Their localization revealed the distal BP3 and BP4 involved in the formation of the junction fragment, between the first and second copy of the CNV. The double orientation of the chimeric reads, mapping on the junction fragments of the triplicated 18q12.1q21.2 region, suggested that the chromosome 18 carrying the CNV has three tandem repeats of 18 Mb, with the middle repeat inverted.

To validate the LRS results, the 18q CNV junction fragments were amplified by PCR assays and sequenced by Sanger Sequencing. The proximal breakpoints, BP1 and BP2, mapped within a 2-bp microhomology sequence (chr18:34,460,465-34,460,466 and chr18:34,494,753-34,494,754, on opposite DNA strands) (Fig. S1A). Sanger Sequencing confirmed BP1 as identified by LRS, whereas BP2 showed a 16-bp shift compared to the LRS-defined breakpoint, at chr18:34,494,753. Visualization of the BP2 region via Integrative Genomics Viewer (IGV) revealed that while the majority of reads indicated a start position at chr18:34,494,769, a smaller subset aligned with the coordinates obtained by Sanger sequencing (Fig. 2A). BP1 is located in an intergenic region between *NOL4* and *DTNA*, whereas BP2 is situated within intron 1 of the *DTNA* gene.

The distal BP3 and BP4 shared a 3-bp microhomology sequence (chr18:52,858,930-52,858,932 and chr18:52,866,374-52,866,376, on opposite strands) (Fig. S1B). While the BP3 position identified by LRS was confirmed, Sanger Sequencing revealed a different position for BP4 compared to the one initially identified by LRS. Specifically, the same sequence identified by LRS was found 80 bp downstream of BP4 (chr18:52,866,294-52,866,373), indicating the presence of a 4.2-kb deletion. Genomic annotation showed that this deletion, along with BP3 and BP4 is located within intron 2 of the *DCC* gene. This intron contains LTR49-int and L1PA7 repetitive elements, as well as several deletions reported in the Database of Genomic Variants (Fig. S2).

Based on the results, we hypothesized that the sequences between BP1 and BP2 and between BP3 and BP4 would be present in three copies, consistent with their lower LRS coverage compared to that detected for the region mapping from BP2 and BP3, which is present in four copies (Fig. S3).

### Potential molecular mechanism generating the 18q12.1q21.2 tetrasomy and the 18q21.2q23 LOH

The generation of the 18q12.1q21.2 triplicated segment requires a three-chromatid exchange, mediated by two events. LRS and Sanger Sequencing allowed us to identify the sequences involved in the two events, which showed 50% homology if aligned in an inverted orientation (Fig. S4), suggesting the potential underlying mechanisms.

**Fig. 3:**
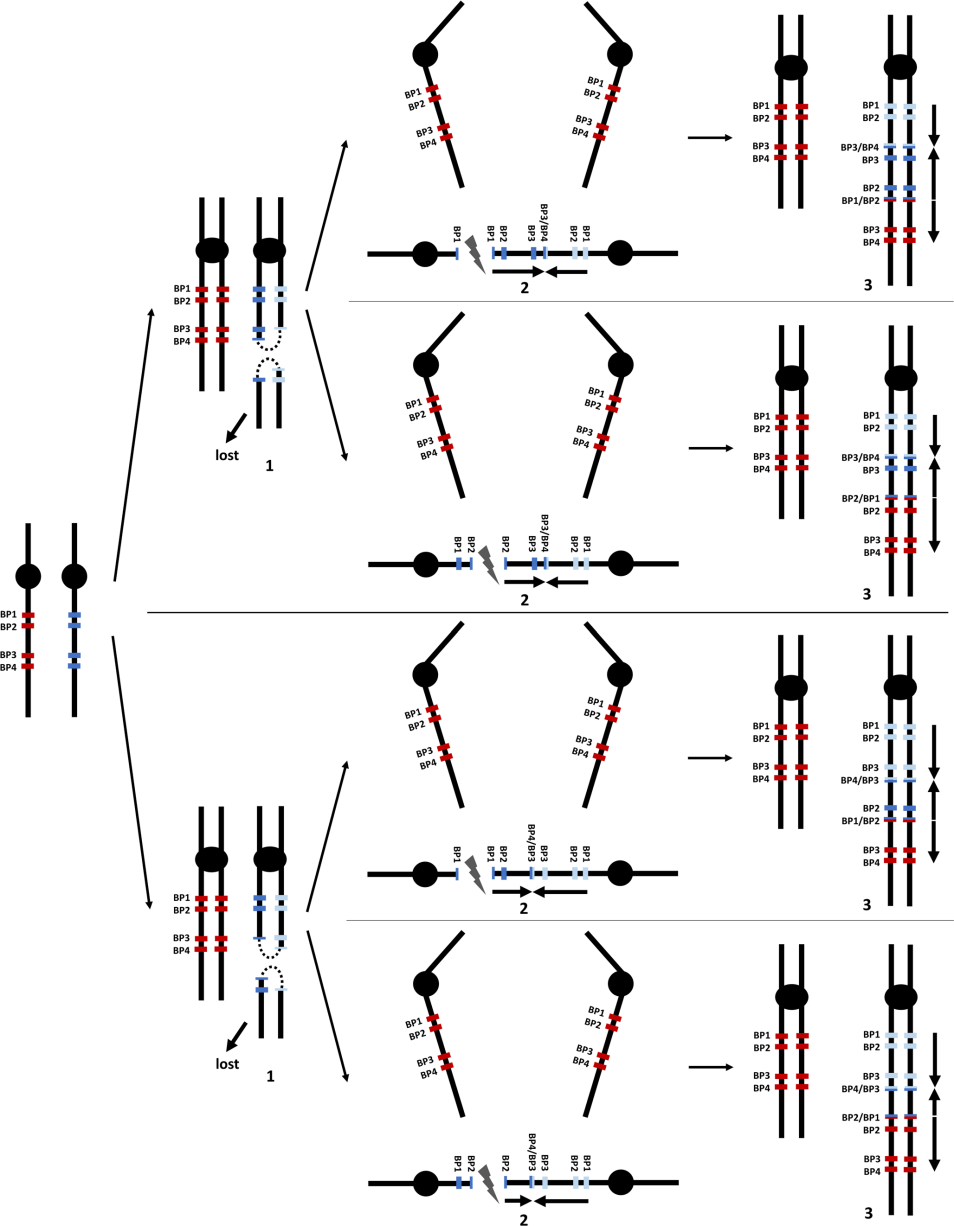
Schematic representation of the proposed mechanism explaining the 18q12.1q21.2 tetrasomy identified in the fetus. Regions including the BPs involved in a three-chromatid exchange mechanism are represented by colored rectangles. A transitory dicentric chromosome 18 and an acentric derivative, which is assumed to be lost, may result from a U-type exchange between sister chromatids (1), involving the distal BP3 and BP4. The dicentric chromosome may have broken at BP1 or BP2 (2) during mitosis, producing a derivative chromosome with a terminal deletion, del(18)(q12.1–qter), which is most likely lost, and a rearranged chromatid bearing the duplicated inverted 18q12.1q21.2 segment. A subsequent telomere capture event (3) would stabilize the broken chromatid with the duplication, resulting in a chromosome harboring three copies of the 18q12.1q21.2 region in a direct-inverted-direct orientation, as indicated by arrows. Specifically, the rearranged chromosome has three copies of the BP2–BP3 interval and two copies of the BP1–BP2 and BP3–BP4 intervals, respectively. However, as the four alternative final conformations show, it is impossible to identify which of the three repeated segments has lost one copy of the BP1–BP2 and BP3–BP4 intervals. A normal chromosome 18 is also expected, in addition to the rearranged chromosome

Our data supported the hypothesis that the fusion of two sister chromatids occurred through a U-type exchange mechanism, likely mediated by microhomology-based mechanisms such as replication fork stalling and template switching and microhomology-mediated break-induced replication (FoSTeS/MMBIR), or microhomology-mediated end-joining (MMEJ), during S-phase. This event, involving the distal BP3 and BP4 sequences, led to the formation of a transient dicentric chromosome and the loss of an acentric derivative (Fig. 3). Following the U-type exchange event, the dicentric chromosome had a mitotic break at either BP1 or BP2 during anaphase, producing two unequal chromatids: one with a significant terminal deletion, which is probably unstable, and another with the duplicated inverted 18q12.1q21.2 segment (Fig. 3). The chromatid containing the duplication was sustained by subsequent telomere capture, resulting in a chromosome 18 with a triplicated 18q12.1q21.2 region organized in a direct–inverted–direct orientation (Fig. 3). Obviously, in addition to the rearranged chromosome, a normal chromosome 18 was also expected.

The modified chromosome exhibited three copies of the ~ 18 Mb region contained between BP2 and BP3 and two copies of the shorter portions BP1–BP2 and BP3–BP4, although it is impossible to determine which of the three repeated segments lost these sequences. In fact, four different conformations for the rearranged chromosome are consistent with the suggested mechanism and the sequencing findings of the junction fragments (Fig. 3).

Lastly, in line with the telomere capture event, both array-CGH (Fig. 1) and LRS (Fig. S5) results revealed a ~ 26 Mb LOH region, distal to the triplicated region, up to the telomere of chromosomes 18.

### Study of 18q12.1q21.2 gene content

Using an in-silico approach, the potential contribution of the genes included in the 18q12.1q21.2 triplicated region to the phenotype of the fetus was examined. The pTriplo score that predicts the probability of triplosensitivity for the protein-coding genes was chosen as predictor of potential intolerance to increased DNA dosage of the 18q12.1q21.2 genes. In addition, possible genotype-phenotype correlations were evaluated using the Online Mendelian Inheritance in Man (OMIM) database. The 18q12.1q21.2 triplicated region involved 58 protein-coding genes. Among them, nine genes (*MAPRE2*, *CELF4*, *SETBP1*, *SMAD2*, *ACAA2*, *MBD1*, *CXXC1*, *SMAD4*, *MEX3C*) showed a pTriplo score ranging from 0.9 to 1, the maximum value associated with the increased dosage intolerance (Fig. S6).

## Discussion

We provide the molecular characterization of an 18q12.1q21. triplication (resulting in a segmental tetrasomy) identified in a fetus with ultrasound abnormalities, which, to our knowledge, has never been described before using LRS. The molecular definition of the breakpoints allowed us to elucidate the possible mechanisms underlying the rearrangement, as well as characterize the chromosomal alteration and establish the extent of the regions involved. Furthermore, the molecular analysis of this specific rearrangement led to the identification not only of the DNA sequences involved, but also of genomic contexts in which these kinds of chromosomal changes occur.

Thanks to the application of LRS, we resolved the structure of this complex rearrangement with multiple breakpoints and defined the orientation of the triplicated segments, enabling us to speculate about the process that led to the abnormal chromosome. Our data support the hypothesis that the fusion of two sister chromatids occurred through a U-type exchange mechanism, occurring in the mitotic S-phase, probably during the morula stage. Given the low homology found between the distal BP3 and BP4 sequences, among the potential mechanisms mediating U-type exchange, FoSTeS/MMBIR and the MMEJ may have led to formation of a transient dicentric chromosome (Koumbaris et al. 2011; Kato et al. 2020). A chromatid with the duplicated inverted 18q12.1q21.2 segment may result from breakage of the dicentric chromosome during mitosis. The creation of the 18q12.1q21.2 triplicated region in a direct-inverted-direct orientation, coupled with the 18q21.2q23 LOH, may have resulted from a telomere capture event that stabilized the transitory chromatid with the duplication (Meltzer et al. 1993).

The fetal phenotype presented a peculiar dissociation from the classic trisomy 18 (Edwards syndrome). While the fetus exhibited specific signs such as congenital diaphragmatic hernia (CDH) and mild facial dysmorphisms (hypertelorism, low-set ears), it notably lacked the hallmark features of full trisomy 18, such as severe intrauterine growth restriction (IUGR), clenched hands with overlapping fingers, and rocker-bottom feet. The full trisomy 18 induces complex, genome-wide gene expression alterations affecting multiple developmental pathways simultaneously (Wang et al. 2022). In contrast, our case involves a segmental tetrasomy of the 18q12.1q21.2 region on a diploid background. However, the comparison with partial duplications of the same chromosomal interval described in the DECIPHER Database further clarifies the crucial role of copy number dosage; nevertheless, it is challenging to compare certain aspects, like intellectual disability or feeding difficulties, because the majority of the cases described are not prenatal. Our case, presenting with a lethal left-sided CDH and neuronal migration defects (heterotopia), suggests that the tetrasomic dosage (4 copies) of genes within this region—particularly those involved in the TGF-beta pathway like *SMAD2* and *SMAD4*—exceeds a critical threshold for diaphragm development, resulting in a more severe, organ-specific phenotype compared to the trisomic state.

One hypothesis is that the specific amplification of proliferation-driving genes (e.g., *SETBP1*, *MAPRE2*) likely conferred a selective clonal advantage in mesenchymal culture, masking the mosaicism observed in cytotrophoblast analysis. In this regard, the discrepancy between the mosaicism detected in cytotrophoblasts and the LRS results is likely due to the cell source. Although LRS has a 5% detection limit, it was performed on DNA from cultured mesenchymal cells where the normal 46,XX cell line was negatively selected during in vitro expansion. This underlines a key limitation in prenatal diagnostics: molecular methods can only detect mosaicism present in the specific cell aliquot analyzed, which may vary significantly between tissues or after culture-induced clonal selection. Meanwhile, the concurrent overexpression of developmental regulators (e.g., *SMAD2* and *SMAD4*) caused a catastrophic failure of diaphragmatic closure, as well as focal defects of corticogenesis in vivo.

Unfortunately, the LRS analysis was not extended to the parents because of the absence of parental consent. Consequently, we were unable to identify the parental origin of the aberrant chromosome 18. For the same reason, it is not possible to completely exclude alternative mechanisms underlying the rearrangement. If the 46,XX cells detected in cytotrophoblast resulted from a vanishing twin, the formation of the rearranged chromosome could be explained by a three-chromatid exchange mechanism occurring during the pre-meiotic S phase or the first meiotic prophase, as illustrated in Fig. S7. Subsequently, a meiotic non-disjunction event involving the normal and rearranged chromosomes may have led to a trisomic condition in the zygote, which was later resolved through trisomic rescue with loss of the chromosome contributed by the normal gamete, consistent with the presence of a terminal LOH region on chromosome 18.

Although currently limited by higher costs, LRS holds great promise for future routine diagnostics by streamlining the workflow into a single analytical step that precisely characterizes structural variant orientation and breakpoints. Moreover, in cases involving LOH, trio-based LRS analysis is pivotal for determining the parental origin of the aberration and evaluating the clinical risks associated with the unmasking of recessive alleles.

## Materials and methods

### Conventional karyotyping

Under a dissecting microscope, the placenta biopsy was separated from maternal decidual tissue and blood clots, and then divided into two aliquots. One aliquot was kept intact to collect spontaneous metaphases after 24 h of incubation and the other one treated with the pronase enzyme to separate mesenchymal cells in order to set up medium-term cultures. The analysis was performed on both samples. The metaphases were analyzed by means of QFQ banding; the images were captured at 100× magnification and they were analyzed by CromoWinPlus software (Tesi Imaging Milano, Italy). The karyotypes were expressed following the guidelines of the International System for Cytogenomic Nomenclature 2024 (McGowan-Jordan et al., 2024).

### Molecular karyotyping

Array-CGH analysis was performed using GenetiSure Cyto 4 × 180 K CGH + SNP Microarray kit with an overall median probe spacing resolution of ~ 44,5 Kb (Agilent Technologies, Palo Alto, CA, USA). Protocols provided by Agilent were followed with no modifications. Genomic DNA was extracted from cultured mesenchymal cells with an automated method by a Maxwell^®^ CSC Instrument IVD (Promega Corporation, Madison, WI, USA) and the maternal contamination was excluded using PowerPlex^®^ 16 HS System kit (Promega Corporation, Madison, WI, USA) according to manifacturer’s instruction. DNA control reference was provided by Agilent. The arrays were scanned at 3-µm resolution using a SureScan DX Microarray scanner Agilent and analyzed using CytoGenomics software v5.3 (Agilent Technologies, Palo Alto, CA, USA) by the aberration detection method 2 (ADM-2) algorithm with a threshold of 5. Putative chromosome copy number changes were defined by intervals of 5 or more adjacent probes and were considered as being duplicated or deleted when results exceeded |0.25|. All nucleotide positions were based on the Human Reference Sequence Assembly, December 2013 GRCh38/hg38 of the UCSC Genome Browser (http://genome.ucsc.edu/, accessed 1 October 2025). All the molecular karyotypes were formulated following the guidelines of ISCN 2024. Copy number variations (CNVs) were evaluated by in silico Databases: DGV (Database of Genomic variants; https://dgv.tcag.ca/dgv/, accessed 1 October 2025), DECIPHER (DatabasE of genomiC varIation and Phenotype in Humans using Ensembl Resources; https://www.deciphergenomics.org/, accessed 1 October 2025) and ClinVar (https://www.ncbi.nlm.nih.gov/clinvar/, accessed 1 October 2025).

### ONT promethion library preparation and sequencing

Genomic DNA extracted from chorionic villus sample was assessed by Qubit (dsDNA HS Quantification Assay), Agilent 4200 TapeStation (Genomic DNA ScreenTape Assay) and NanoDrop Spectrophotometer prior to library preparation. A sequencing library was prepared starting from 1 µg of gDNA using the ligation sequencing kit SQK-LSK114 (Oxford Nanopore Technologies, ONT, Oxford, UK) following the manufacturer’s instructions. Five hundred femtomoles of the final library were loaded onto a PromethION2 Solo flow cell (R10.4.1) and the data were collected for 3 days.

### Bioinformatics analysis

Sequencing data were acquired in POD5 format using MinKNOW (25.03.9 version) and base-calling was performed using Dorado (v0.9.0) with the model dna_r10.4.1_e8.2_400bps_sup@v4.3.0 (https://github.com/nanoporetech/dorado, accessed 1 June 2025). The resulting FASTQ reads were aligned to the human reference genome (GRCh38) using minimap2 (Li et al. 2018). Coverage profiles were computed using mosdepth (Pedersen et al. 2018), and variant calling was performed with default parameters using Clair3 for SNPs and small indels, Spectre for copy number variation (CNV) analysis and Sniffles2 for structural variant (SV) detection (Epi2me. [(accessed on 1 June 2025)], available online: https://github.com/epi2me-labs/wf-human-variation. Candidate variants were manually visualized using the Integrative Genomics Viewer (IGV, v2.18.0) (Robinson et al. 2011).

### PCR and sequencing analysis

Polymerase chain reactions (PCRs) were performed on gDNA extracted from chorionic villus sample using GoTaq^®^ G2 DNA Polymerase (Promega, Fitchburg, WI, USA), according to the standard procedures. The oligonucleotides designed for the amplification of the junction fragments of 18q12.1q21.2 triplicated region are shown in Supplementary Table S2. The PCR products were sequenced through the Terminator v3.1 Cycle Sequencing Kit (Thermo Fisher) and resolved on an automated ABI-3130xl DNA genetic analyzer (Thermo Fisher). The sequencing results were analyzed using Chromas Lite software (Technelysium Pty Ltd, South Brisbane, Australia).

## Supplementary Information

Below is the link to the electronic supplementary material.

Supplementary Material 1

Supplementary Material 2

Supplementary Material 3

Supplementary Material 4

Supplementary Material 5

Supplementary Material 6

Supplementary Material 7

Supplementary Material 8

Supplementary Material 9

Supplementary Material 10

## Data Availability

All data needed to evaluate the conclusions in the paper are present in the paper and/or the Supplementary Materials. All relevant data are available from the authors upon request. Sequencing data are restricted due to ethical considerations and are not deposited on public platforms.

## References

[CR1] Bourgois A, Bizaoui V, Colson C, Vincent-Devulder A, Molin A, Gérard M, Gruchy N (2024) Phenotypic and genotypic characterization of 1q21.1 copy number variants: A report of 34 new individuals and literature review. Am J Med Genet A 194(3):e63457. 10.1002/ajmg.a.63457 37881147 10.1002/ajmg.a.63457

[CR2] Carvalho CM, Lupski JR (2016) Mechanisms underlying structural variant formation in genomic disorders. Nat Rev Genet 17(4):224–238. 10.1038/nrg.2015.25 26924765 10.1038/nrg.2015.25 PMC4827625

[CR3] Carvalho CM, Zhang F, Liu P, Patel A, Sahoo T, Bacino CA, Shaw C, Peacock S, Pursley A, Tavyev YJ et al (2009) Complex rearrangements in patients with duplications of MECP2 can occur by fork stalling and template switching. Hum Mol Genet 18(12):2188–2203. 10.1093/hmg/ddp151 19324899 10.1093/hmg/ddp151 PMC2685756

[CR4] Carvalho CM, Ramocki MB, Pehlivan D, Franco LM, Gonzaga-Jauregui C, Fang P, McCall A, Pivnick EK, Hines-Dowell S, Seaver LH et al (2011) Inverted genomic segments and complex triplication rearrangements are mediated by inverted repeats in the human genome. Nat Genet 43(11):1074–1081. 10.1038/ng.944 21964572 10.1038/ng.944 PMC3235474

[CR5] Emiliani FE, Ismail AAO, Hughes EG, Tsongalis GJ, Zanazzi GJ, Lin CC (2025) Nanopore-based random genomic sampling for intraoperative molecular diagnosis. Genome Med 17(1):6. 10.1186/s13073-025-01427-7 39833913 10.1186/s13073-025-01427-7 PMC11744943

[CR6] Grochowski CM, Bengtsson JD, Du H, Gandhi M, Lun MY, Mehaffey MG, Park K, Höps W, Benito E, Hasenfeld P et al (2024) Inverted triplications formed by iterative template switches generate structural variant diversity at genomic disorder loci. Cell Genom 4(7):100590. 10.1016/j.xgen.2024.100590 38908378 10.1016/j.xgen.2024.100590 PMC11293582

[CR7] Kato T, Inagaki H, Miyai S, Suzuki F, Naru Y, Shinkai Y, Kato A, Kanyama K, Mizuno S, Muramatsu Y, Yamamoto T, Shinya M, Tazaki Y, Hiwatashi S, Ikeda T, Ozaki M, Kurahashi H (2020) The involvement of U-type dicentric chromosomes in the formation of terminal deletions with or without adjacent inverted duplications. Hum Genet 139(11):1417–1427. 10.1007/s00439-020-02186-8 32488466 10.1007/s00439-020-02186-8

[CR8] Koumbaris G, Hatzisevastou-Loukidou H, Alexandrou A, Ioannides M, Christodoulou C, Fitzgerald T, Rajan D, Clayton S, Kitsiou-Tzeli S, Vermeesch JR et al (2011) FoSTeS, MMBIR and NAHR at the human proximal Xp region and the mechanisms of human Xq isochromosome formation. Hum Mol Genet 20(10):1925–1936. 10.1093/hmg/ddr074 21349920 10.1093/hmg/ddr074 PMC3428953

[CR9] Li H (2018) Minimap2: pairwise alignment for nucleotide sequences. Bioinformatics 34(18):3094–3100. 10.1093/bioinformatics/bty191 29750242 10.1093/bioinformatics/bty191 PMC6137996

[CR10] McGowan-Jordan J, Hastings RJ, Moore S (eds) (2024) ISCN 2024: An international system for human cytogenomic nomenclature (2024). Karger 10.1159/000516655 34407535

[CR11] Meltzer PS, Guan XY, Trent JM (1993) Telomere capture stabilizes chromosome breakage. Nat Genet 4(3):252–255. 10.1038/ng0793-252 8358433 10.1038/ng0793-252

[CR12] Pedersen BS, Quinlan AR (2018) Mosdepth: quick coverage calculation for genomes and exomes. Bioinformatics 34(5):867–868. 10.1093/bioinformatics/btx699 29096012 10.1093/bioinformatics/btx699 PMC6030888

[CR13] Pelgrims E, Lynch SA, Hannes L, Hoffer MJV, Melotte C, Van Haeringen A, Swillen A, Breckpot J (2023) Triplications of chromosome 1p36.3, including the genes GABRD and SKI, are associated with a developmental disorder and a facial gestalt. Am J Med Genet A 191(7):1889–1899. 10.1002/ajmg.a.63222 37129290 10.1002/ajmg.a.63222

[CR14] Robinson JT, Thorvaldsdóttir H, Winckler W, Guttman M, Lander ES, Getz G, Mesirov JP (2011) Integrative genomics viewer. Nat Biotechnol 29(1):24–26. 10.1038/nbt.1754 21221095 10.1038/nbt.1754 PMC3346182

[CR15] Showpnil IA, Hernandez Gonzalez E, Ramadesikan M, Marhabaie S, Daley M, Dublin-Ryan A, Pastore L, Gurusamy MT, Hunter U, Stone JM, B. S., et al (2024) Long-read genome sequencing resolves complex genomic rearrangements in rare genetic syndromes. NPJ Genom Med 9(1):66. 10.1038/s41525-024-00454-4 39695126 10.1038/s41525-024-00454-4 PMC11655636

[CR16] Wang J, Reddy KS, Wang E, Halderman L, Morgan BL, Lachman RS, Lin HJ, Cornford ME (1999) Intrachromosomal triplication of 2q11.2-q21 in a severely malformed infant: case report and review of triplications and their possible mechanism. Am J Med Genet 82(4):312–317. 10.1002/(sici)1096-8628(19990212)82:4<312::aid-ajmg7>3.0.co;2-9 10051164 10.1002/(sici)1096-8628(19990212)82:4<312::aid-ajmg7>3.0.co;2-9

[CR17] Wang J, Chen Z, He F, Lee T, Cai W, Chen W, Miao N, Zeng Z, Hussain G, Yang Q, Guo Q, Sun T (2022) Single-cell transcriptomics of cultured amniotic fluid cells reveals complex gene expression alterations in human fetuses with trisomy 18. Front Cell Dev Biol 10:825345. 10.3389/fcell.2022.825345 35392164 10.3389/fcell.2022.825345 PMC8980718

