# Supplementary figures and images for: Long-read genome sequencing resolves a de novo complex 18q12.1q21.2 triplication causing partial tetrasomy and reveals its underlying mechanism
Source: Hum Genet. 2026 Jul 4;145(1):56. doi: 10.1007/s00439-026-02855-0 (PMC13332952; doi:10.1007/s00439-026-02855-0)

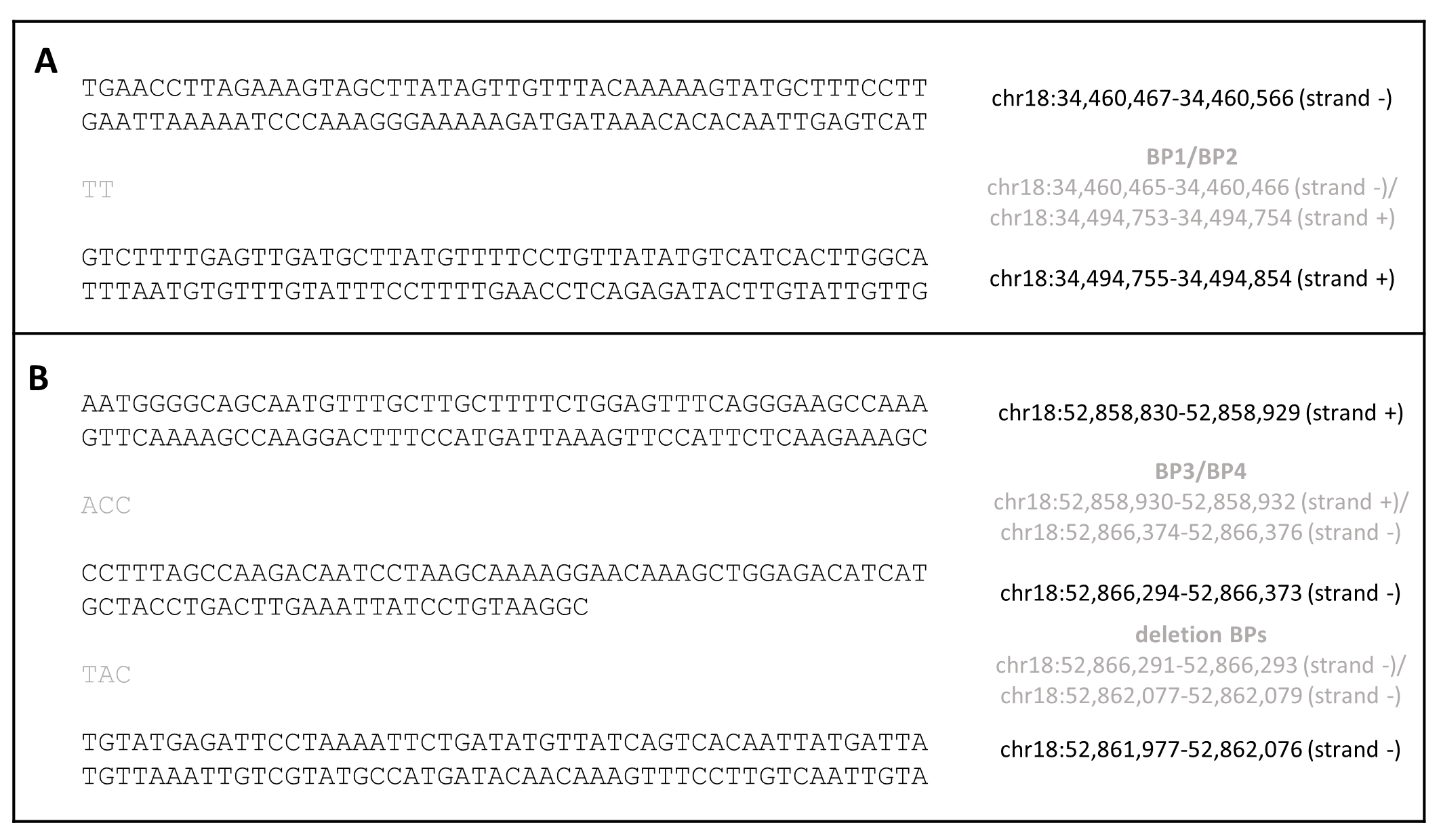

Supplement: Supplementary file 3 — Supplementary Material 3 [file 439_2026_2855_MOESM3_ESM.tif]

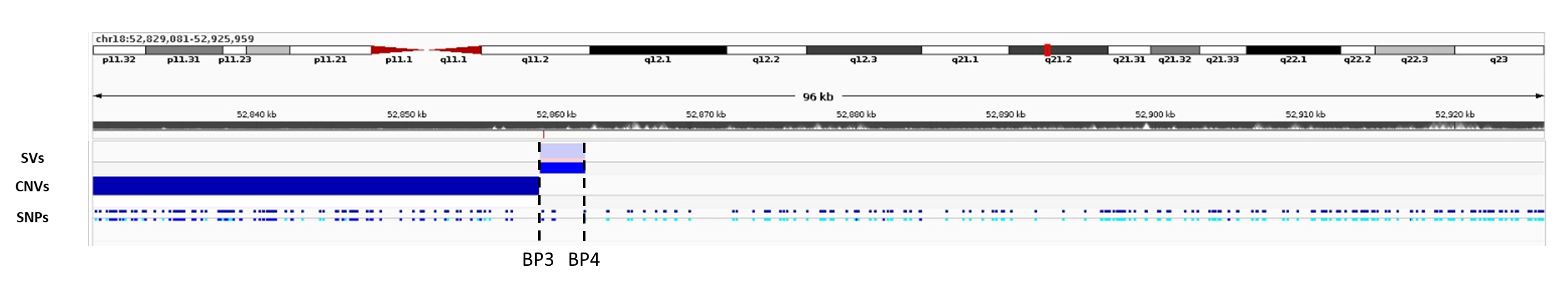

Supplement: Supplementary file 4 — Supplementary Material 4 [file 439_2026_2855_MOESM4_ESM.tif]

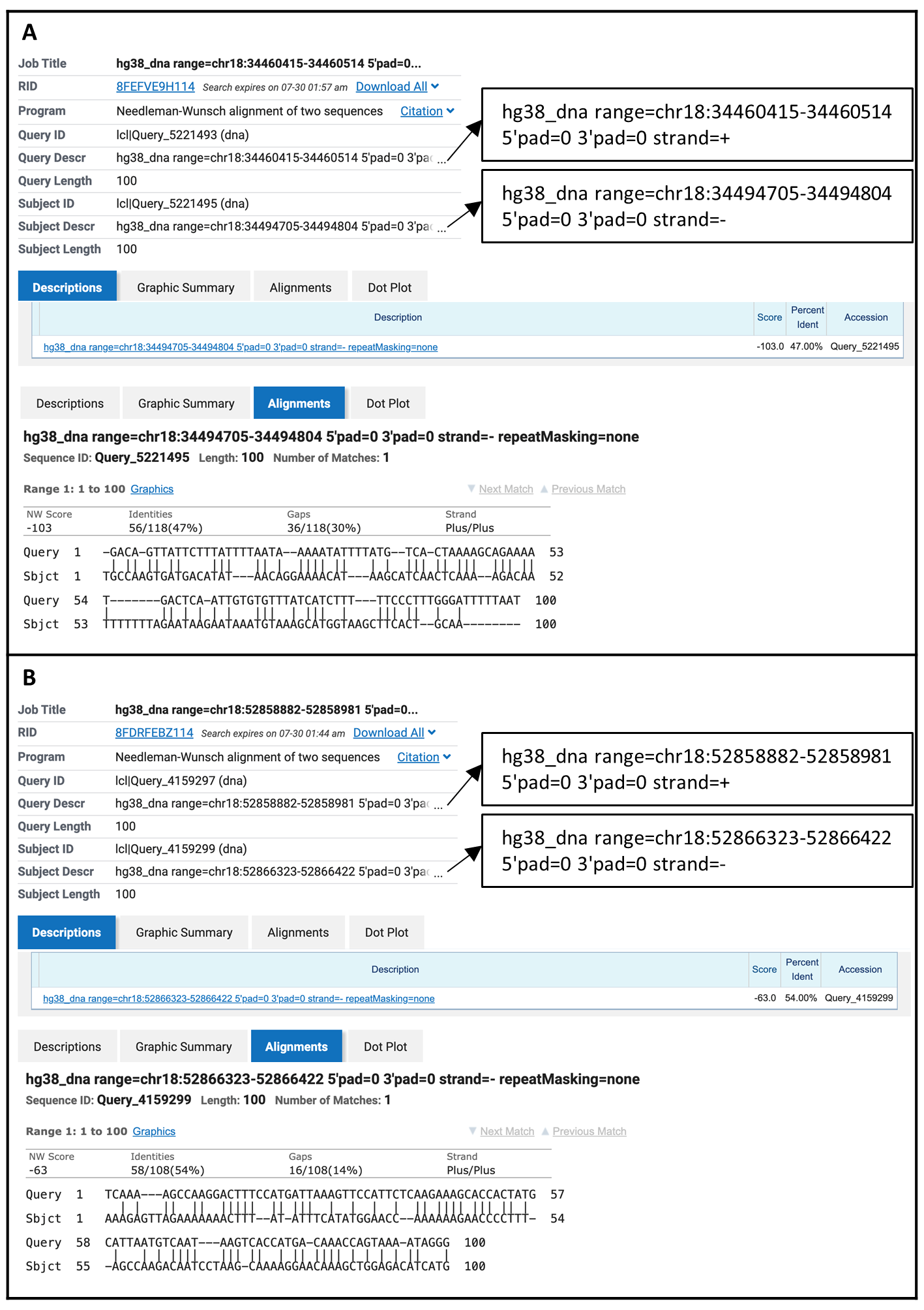

Supplement: Supplementary file 5 — Supplementary Material 5 [file 439_2026_2855_MOESM5_ESM.tif]

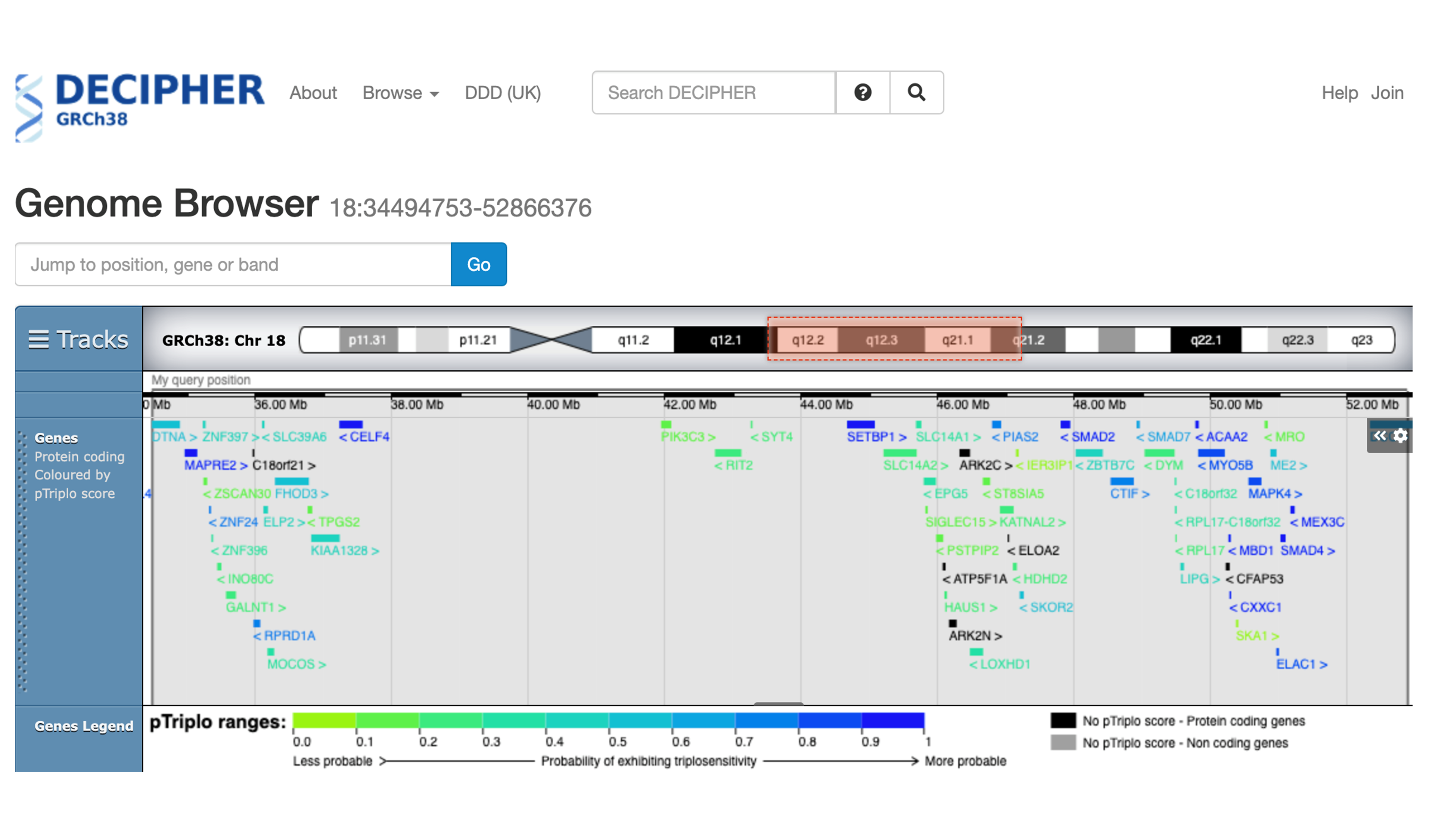

Supplement: Supplementary file 6 — Supplementary Material 6 [file 439_2026_2855_MOESM6_ESM.tif]

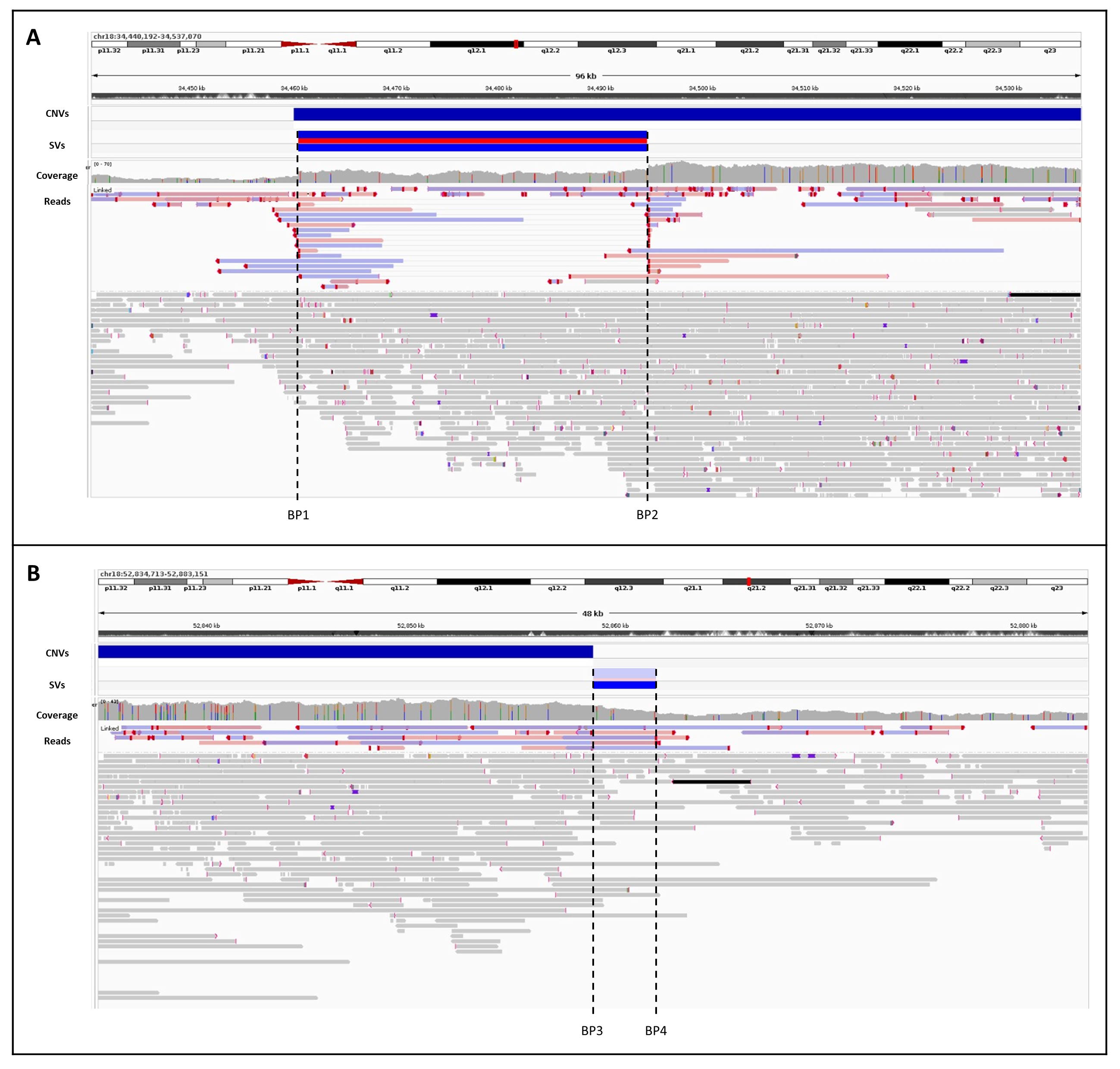

Supplement: Supplementary file 7 — Supplementary Material 7 [file 439_2026_2855_MOESM7_ESM.tif]

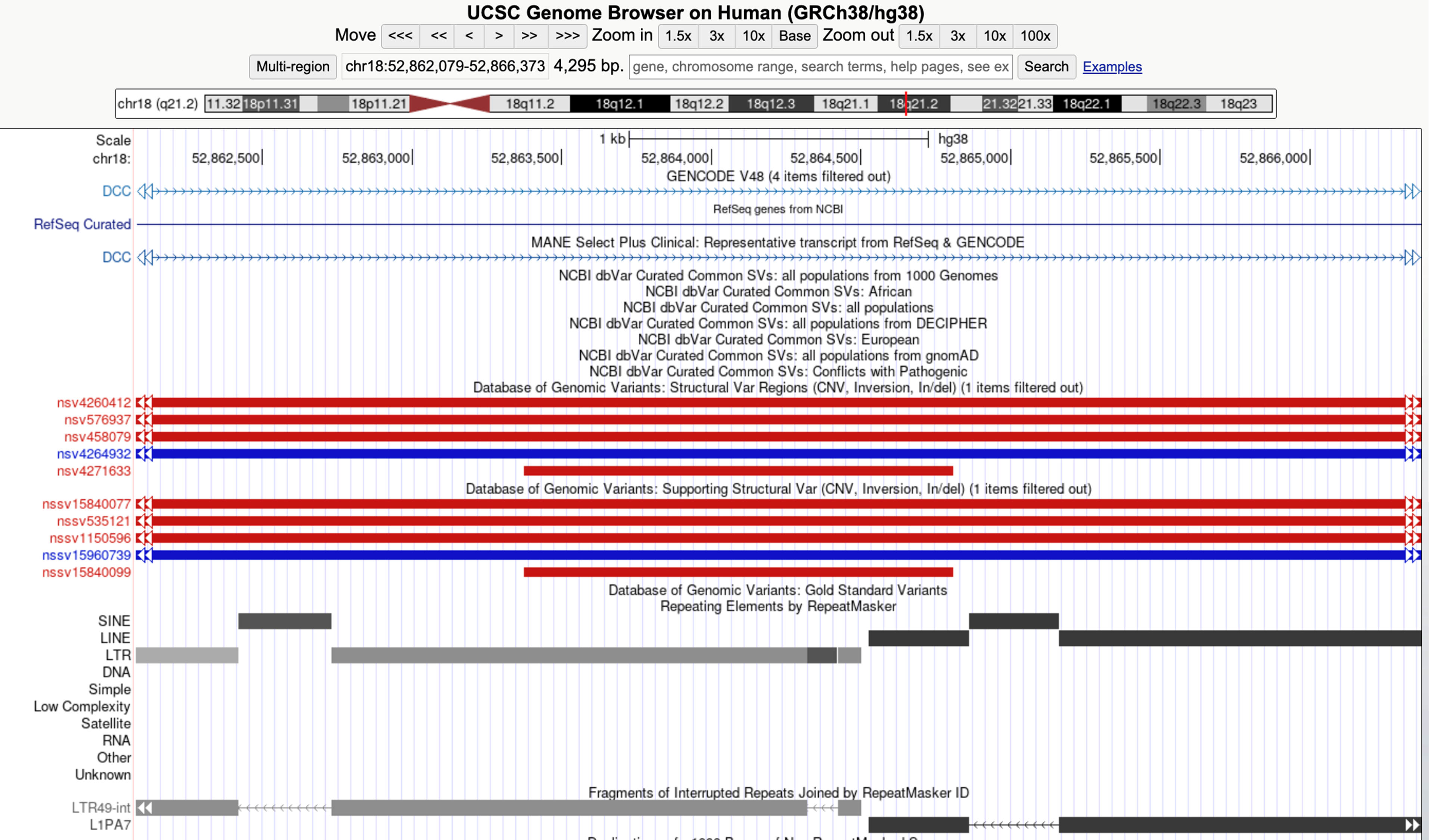

Supplement: Supplementary file 8 — Supplementary Material 8 [file 439_2026_2855_MOESM8_ESM.tif]

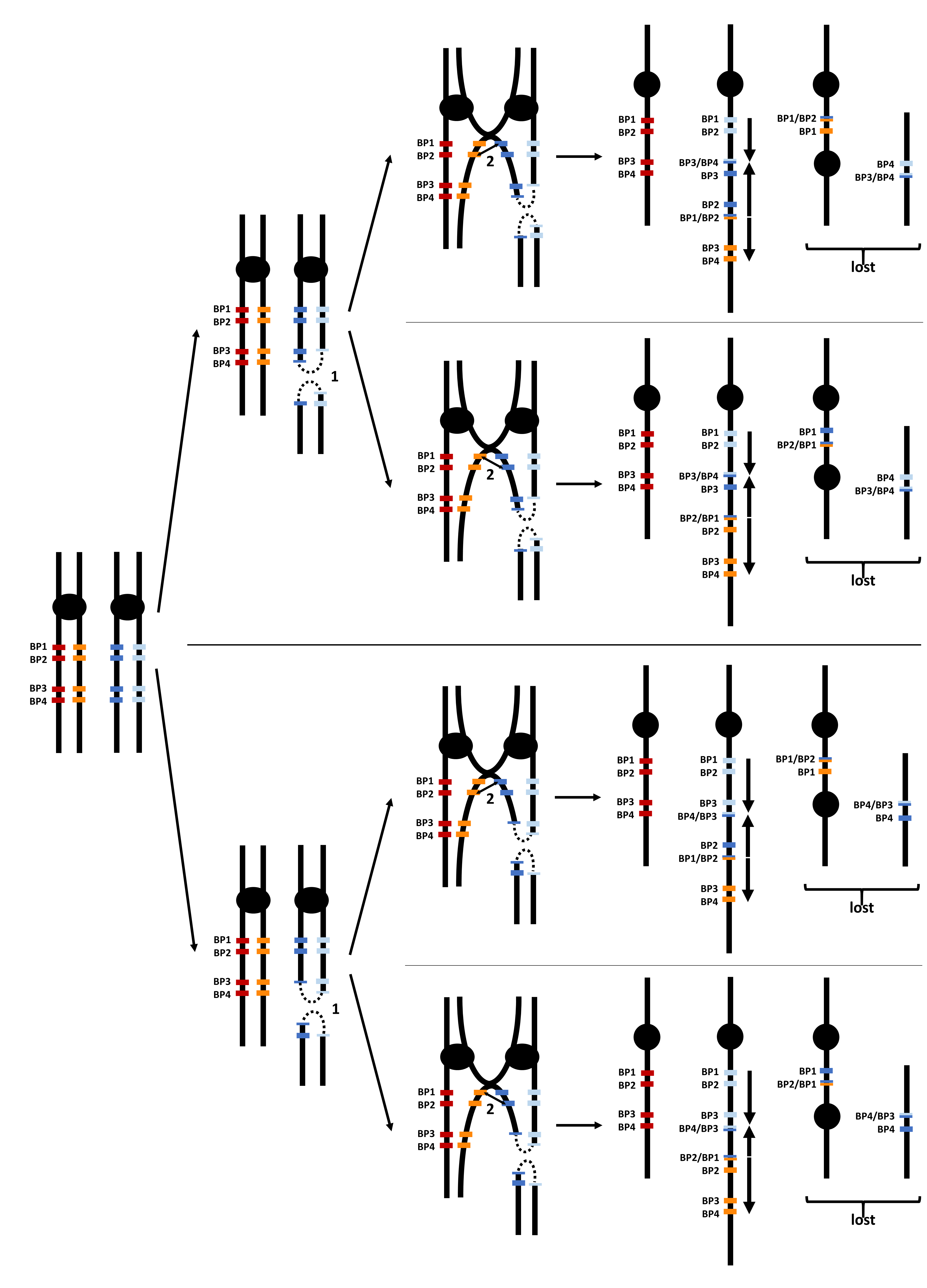

Supplement: Supplementary file 10 — Supplementary Material 10 [file 439_2026_2855_MOESM10_ESM.tif]
