# Supplementary material for: Long-read genome sequencing resolves a de novo complex 18q12.1q21.2 triplication causing partial tetrasomy and reveals its underlying mechanism
Source: Hum Genet. 2026 Jul 4;145(1):56. doi: 10.1007/s00439-026-02855-0 (PMC13332952; doi:10.1007/s00439-026-02855-0)
Supplement: Supplementary file 9 — Supplementary Material 9 [file 439_2026_2855_MOESM9_ESM.docx]

**Legends to supplementary figures**

Fig. S1. Characterization of the 18q CNV junction fragments by Sanger sequencing. **A:** Sequence of the junction fragment including the proximal BP1 and BP2. B: Sequence of the junction fragment involving the distal BP3 and BP4. Interestingly, a 4.2 kb deletion has been detected within the BP3-BP4 junction fragment.

Fig. S2. Structural variants and repeat elements mapping within the 18q12.1q21.2 region. As shown by UCSC Genome Browser, several copy number loss/deletions (in red) and copy number gain/duplications (in blue) are listed in Database of Genomic Variants within this genomic region, such us repeat elements (in gray).

Fig. S3. LRS coverage profiles detected in the BPs’ regions of the 18q CNV. The Integrative Genomics Viewer (IGV) visualization shows CNVs, structural variants (SVs), coverage profiles and reads alignments around the 18q CNV BPs. Greater coverage than that of the 2-copy region and less than that of the 4-copy region seems to be present between BP1 and BP2 (**A**) and between BP3 and BP4 (**B**).

Fig. S4. Alignment analysis of the sequences that mediated the generation of the 18q12.1q21.2 triplicated segment. As shown by BLAST tool, sequence homology of 47% and 54% was found comparing BP1 vs BP2 (**A**) and BP3 vs BP4 (**B**) sequences, respectively.

Fig. S5. LOH region identified by LRS. The IGV visualization shows SNP calls (highlighted in blue) and below them their homozygous (light blue) or heterozygous (blue) status. Until the end of the 18q CNV, both homozygous and heterozygous SNPs are present, while the LOH region begins after the triplicated 18q12.1q21.2 segment.

Fig. S6. Gene content of 18q12.1q21.2 triplicated region. As shown by Decipher Database, the patient CNV involved 58 protein-coding genes. Among these genes, 9 are sensitive to gene dosage, showing a pTriplo score greater than 0.9.

Fig. S7. Schematic representation of the alternative mechanism, based on a meiotic error, to explain the 18q12.1q21.2 tetrasomy identified in the fetus. The regions including the BPs involved in a three-chromatid exchange mechanism are represented by colored rectangles. A U-type exchange between sister chromatids (1), involving the distal BP3 and BP4, could lead to the formation of a transient dicentric chromosome 18 and an acentric derivative, which is presumed to be lost. A second event, mediated by MMEJ between homologous chromatids (2), at the proximal BP1 and BP2, would have allowed the formation of the chromosome carrying three copies of the 18q12.1q21.2 region in a direct-inverted-direct orientation, as shown by arrows, and a small inv dup(18) marker chromosome, presumed to be lost. Precisely, three copies for the sequence included between BP2 and BP3 and two copies for the regions BP1–BP2 and BP3–BP4 are expected on the rearranged chromosome. Nevertheless, it is not possible to establish which of the three repeated segments lost a copy of the BP1–BP2 and BP3–BP4 regions, as shown by the four possible final conformations. In addition to the rearranged chromosome, a normal chromosome is also expected.
